# Determinants of health facility delivery among women in Tharaka Nithi county, Kenya

**DOI:** 10.11604/pamj.supp.2016.25.2.10273

**Published:** 2016-11-26

**Authors:** Eliphas Gitonga, Felarmine Muiruri

**Affiliations:** 1 Public Reproductive Health, Kenyatta University; 2 Public and Reproductive Health, Amref Health Africa

**Keywords:** Health facility delivery, maternal determinants, institutional delivery

## Abstract

**Introduction:**

Kenya records a high maternal mortality ratio 362 maternal deaths per 100 000 live births. Tharaka Sub County has poor transport infrastructure, low levels of socio-economic status and long distances to health facilities. Secondary to these factors, delivering in a health facility is a challenge. Delivering in a health facility is one of the strategies to avert maternal death through skilled birth attendance. The aim of the study was to evaluate the determinants of health facility delivery among women in Tharaka Nithi County.

**Methods:**

The study design was descriptive cross sectional survey. Semi structured questionnaires were used for data collection. Stratified sampling was used to select the facilities. Systematic sampling was used to select the respondents. The sample size was 345. Descriptive statistics, chi square, Fishers exact and logistic regression were used in analysis.

**Results:**

Majority (79%) of the respondents delivered in health facilities. Health facility deliveries were highest (80%) among women aged 20-34 years and among those who attended level 4 facilities for ante natal care (88.3%). Health facility deliveries were lowest among women with five or more births. Health facility deliveries were higher among those who attended at least 4 ante natal visits (87.2%) and having individual birth plans (90%). The likelihood of health facility deliveries was increased by increase in level of education (1.6 times), household income (2.4 times), attending a higher level facility ante natally (1.4 times), birth preparedness (3 times), attending at least 4 ante natal visits (2.9 times) but was decreased by an increase in parity (0.5 times).

**Conclusion:**

The determinants of place of delivery are maternal age, level of education, household income, parity, attendance of ante natal care four or more times and birth preparedness.

## Introduction

The health status of mothers and children is an important indicator of the overall economic health and well-being of a country. Maternal health is inextricably linked with the survival of newborns. For every woman who dies, another 30 suffer long-lasting injuries and illnesses such as obstetric fistula [1]. Globally, it is estimated that 10.7 million died in the 25 years between 1990 and 2015 due to maternal causes [2].

The maternal mortality is high in many countries to a point that in every minute a woman dies due to pregnancy related complications. Developing countries account for 99% of the global maternal deaths with sub-Saharan Africa region alone accounting for 62% [2]. Maternal mortality is a major public health problem in Kenya. According to Kenya demographic and health survey 2014/2015, the maternal mortality ratio is 362 deaths per 100,000 live births. A ratio equal to or above 300 maternal deaths per 100 000 live births is considered high. Despite 95.5% of ante natal care attendance by health professionals, only 61.2% of deliveries occur in health facilities [1]. In Tharaka Nithi County, where Tharaka sub-county is located, 77.7 % of the women deliver in health facility [3]. In the contrary in Tharaka sub-county, 61 % of the women in the sub county deliver without skilled attendance [4]. Tharaka sub-county also has a problem in accessing health facilities for delivery or emergency care some residents have to travel up to 30 km to reach a health facility for delivery. The area is characterised with poor road network, long distances to health facilities and low socio economic status. Pregnant women with complications face worse situations because the district hospital does not have an operational theatre for caesarean sections or any other obstetric operation [5]. Health facility reduces maternal deaths through skilled birth attendance. Skilled birth attendance is where a pregnant woman is attended by a qualified health worker in a facility that has the requisite resources during delivery. Proper medical attention and hygienic conditions during delivery reduce the risk of complications, infections, or death of the mother and the baby [3]. This study evaluated the determinants of health facility delivery among women in Tharaka sub-county in rural Kenya.

## Methods

**Study design and target population:** a descriptive cross-sectional survey design was used. This design is a snapshot approach. It cannot elucidate cause effect relationship. The target population was women who had delivered within one year prior to the study. This was estimated to be 4,732 [6].

**Setting:** the study was conducted in Tharaka sub-county. The area is in Tharaka Nithi County, Kenya. Most of the sub-county is rural. The sub-county covers an area of 1569.5 km2 [5]. It had a total population of 130,098 people among them 67,211 are women [6]. The sub-county has one sub-county hospital, one mission hospital, one sub sub-county hospital, two health centres and twenty dispensaries [5]. The number of women of reproductive age is 31,547. The estimated number of pregnant women is 4732 [6]. The average distance to the nearest facility is 7km. Most of the sub-county does not have good transport network [5]. Tharaka Nithi County meets the ministry of health (Kenya) criteria for areas that have community and institutional risks to maternal health. They include long distance to health facilities, low socio-economic status and poor transport infrastructure. This formed the part of the criteria of selecting the area of study.

### Variables

The dependent variable was health facility delivery that was dichotomised has health facility delivery or home delivery. The independent variables were age in years, type of employment, level of health facility attended for ante natal care, distance to a facility that can do operative (caesarean section) delivery, gravidity, parity, history of still births, antenatal care attendance and birth preparedness.

### Sampling, data collection and data analysis

Simple random sampling was used to select Tharaka from the three sub-counties that make Tharaka Nithi County. Stratified sampling was used to select nine health facilities from a total of fourteen in the sub-county. Stratification of the health facilities was done using the level of service delivery as classified by Kenya Essential Package for Health and the region where the facility falls. Systematic sampling was used where every 14th client attending maternal/child health clinic in the sampled facilities was interviewed. The calculation is as follows Kth =N/n. N is the target population which is 4732, n is the sample size which is 345. Kth =4732/345 =14. The first respondent was picked by randomly picking the attendance number that the mothers were given when they arrived at the clinic. Sample size of 345 was calculated by formula as used by Kothari (2004). Semi-structured questionnaires were used to collect data. Pre test was done in Kajuki health centre which has similar characteristics with the area of study. Data collection was carried out between February to July 2014.

Data was entered into STATA version 11. Descriptive statistics and Chi Square at 95% confidence interval was used to test the association of the independent and dependent variables. The variables that had statistically significant association using chi square and Fishers exact were subjected to logistic regression to generate the odds ratios. All results were interpreted as significant if p<0.05.

### Inclusion and exclusion criteria

Participants were included in the study if they women who had delivered within one year prior to the study period. They were also supposed to have been willing to consent voluntarily to participate. Women who were critically sick and mentally unwell were excluded because they were deemed ethically unable to consent.

### Ethical considerations

Ethical approval was sought from Kenyatta University Ethics and review committee and research permit from Ministry of Higher Education Science and Technology to carry out the study. Permission was sought from Tharaka sub-county health management team. Informed consent was sought from each respondent. Participation was voluntary. The participants were given a consent form to sign before participation.

## Results

### Demographic and institutional factors associated with health facility delivery

Majority (79%) of the respondents delivered in health facilities. The objective of this analysis was to assess the association between the health facility delivery and demographic and institutional factors. The proportion of women delivering in health facilities was highest among ages 20-34 years (above 80%) and lowest between ages 40-44 years (54%). There was a significant association between age group and health facility delivery (p<0.001). The proportion of women who delivered in health facilities were higher among those with formal employment (100%) compared to those in non-formal employment. There was a statistically significant association between type of employment and health facility delivery (Fishers exact =0.017).

Majority (88.3%) of respondents who attended level four facilities for ante natal care delivered in health facilities compared to those who attended level two and three facilities. There was a statistically significant association the level of ante care facility and health facility delivery (p=0.004). Table 1 shows the demographic and institutional factors associated with place of delivery.

**Table 1:** Demographic and institutional factors associated with place of delivery

Variable	Group	Home	Health facility	Statistical values
Age in years in groups N= 345	Below 20	9 (28.1%)	23 (71.9%)	Chi2 (5) = 27.64^++^ P <0.001
	20-24	9 (8.7%)	95(91.3%)	
	25-29	16(19.8%)	65 (80.2%)	
	30-34	11 (19.3%)	46 (80.7%)	
	35-39	18(38.3%)	29(61.7%)	
	40-44	11(45.8)	13(54.2%)	
Type of employment of the respondent N=345	Non formal	74 (22.6%)	253 (77.4%)	Fishers’ exact = 0.017^+^
	Formal	0 (0%)	18 (100%)	
Level of facility attended N=327	Level 2	14 (19.7%)	57 (80.3%)	Chi2 (2) = 10.888^+^ P= 0.004
	Level 3	24 (28.2%)	61 (71.8%)	
	Level 4	20(11.7%)	151(88.3%)	
Distance to operative facility N= 345	5km and less	24 (45.3%)	29 (54.7%)	Chi2 (1) = 21.11^++^ P<0.001
	More than 5km	50 (17.1%)	242 (82.9%)	

### Maternal factors associated with health facility delivery

The objective of this analysis was to assess the association between the health facility delivery and maternal factors. The proportion of women who delivered in health facilities was lowest (55.2%) among women with five or more pregnancies. There was a significant statistical association between number of pregnancies (gravida) and place of delivery (p<0.001). The women with five or more births had the lowest proportion (57.6%) of health facility deliveries. There was a significant statistical association between number of parity and place of delivery (p<0.001). A higher proportion of women who had attended four or more visits as recommended in focused antenatal delivered in health facilities (87.15%) compared to those who attended less than four visits (69%). The proportion of women who delivered in the health facilities was higher among women who had attended ante natal visits in first and second trimesters, 84.6% and 87.5% respectively. There was a significant statistical association between trimester of first ante natal care attendance and place of delivery (p<0.001). The proportion of women who delivered in health facilities was higher among women who had individual birth plans (90%) than those who did not have (75.6%). Table 2 shows the maternal factors associated with place of delivery.

**Table 2:** Maternal factors associated with place of delivery

Variable	Group	Home	Health facility	Statistical values
Gravida	1-2	30 (15.7%)	161 (84.3%)	Chi2 (2) = 26.568^++^ P<0.001
	3-4	14 (16%)	73 (84%)	
	5+	30 (44.8%)	37 (55.2%)	
Parity	1-2	31 (15.8%)	165 (84.2%)	Chi2 (2) =28.77^++^ P<0.001
	3-4	14 (16%)	73 (84%)	
	5+	29 (46.8%)	33 (53.2%)	
Still birth	No	60 (19.2%)	252 (80.8%)	Chi2 (1) = 9.52^+^ P= 0.002
	Yes	14 (42.4%)	19 (57.58%)	
Attendance of at least 4 ante natal visits	No	51 (30.7%)	115 (69.3%)	Chi2 (1) = 16.33^++^ P<0.001
	Yes	23 (12.85%)	156 (87.15%)	
Trimester of first ANC attendance N= 327	Third	25 (34.25%)	48 (65.75%)	Chi2 (2) = 17.736^++^ P<0.001
	Second	27 (12.5%)	188 (87.5%)	
	First	6 (15.4%)	33(84.6%)	
Birth preparedness N =345	Unprepared	67 (24.4%)	208 (75.6%)	Chi2 (1)= 6.832^+^ P= 0.009
	Prepared	7 (10%)	63 (90%)	

### Demographic and institutional determinants of place of delivery

The objective of this analysis was to assess the demographic and institutional factors that can predict health facility delivery. This was done using logistic regression. An increase in the age of women reduces the likelihood of health facility delivery by 0.7 times. An increase in the level of education of the women increases their likelihood of delivering in health facilities by 1.6 times. An increase in household income increases the likelihood of delivering in health facilities by 2.4 times. Attending a higher level of facility during ante natal care increases the likelihood of delivering in health facilities by 1.4 times. Table 3 shows the demographic and institutional determinants of place of delivery.

**Table 3:** Demographic and institutional determinants of place of delivery

Variable	Odds ratio	P value	Confidence intervals
Age group	0.709^++^	<0.001	0.584 - 0 .845
Level of education	1.646^++^	<0.001	1.249 - 2.171
Occupation of the respondent	0.978	0.826	0.806 - 1.187
Household income	2.374^++^	<0.001	1.551 - 3.634
Level of facility attended during ANC	1.44+	0.036	1.024 - 2.028

+ Significant

### Maternal determinants of place of delivery

The objective of this analysis was to assess the maternal factors that can predict health facility delivery. This was done using logistic regression. An increase in pregnancies reduces the likelihood of delivering in health facility by about 0.5 times. An increase in parity reduces the likelihood of health facility deliveries by about 0.5 times. Attendance of four or more ante natal visits increases the likelihood of health facility delivery by 3 times. Early attendance of ante natal care increases the likelihood of delivering in health facility by 2.3 times. Birth preparedness increases the likelihood of health facility delivery by 2.9 times. Table 4 shows the maternal determinants of place of delivery.

**Table 4:** Maternal determinants of place of delivery

Variable	Odds ratio	P value	Confidence interval
Gravida	0.496^++^	<0.0001	0.361 - 0.682
Parity	0.485^++^	<0.0001	0.352 - 0.669
Attendance of at least 4 ANC visits	3.008^++^	<0.0001	1.738 - 5.203
Trimester of first ANC attendance N= 327	2.329^++^	0.001	1.391 - 3.900
Birth preparedness N =345	2.899+	0.012	1.266 - 6.635

+ Significant

## Discussion

The results indicated that most of the respondents delivered in health facilities. This is contrary to a study in West Pokot County, Kenya [7] that found that the health facility deliveries were at 33% and that by studies done in Tanzania that found it to be 54% [8]. This may be explained by the difference in the characteristics of the areas of study. For example west Pokot has fewer health facilities and poorer transport infrastructure than Tharaka Nithi County. This finding is also higher than the national figure which is 61% [3]. The prevalence of facility deliveries was much lower in Ethiopia at 4.1% [9]. The results indicated that with an increase in age women reduces the likelihood to deliver in health facilities. These results are similar to other studies. Younger women aged between 15 to 19 and those over 35 years, are at greater risk during childbirth. Available literature shows that women of 35 years and over who have more than three children are less likely to use skilled birth attendance during pregnancies [10]. One of the important demographic variables that affects the utilization of health seeking behaviour is mothers’ age at the time of birth. Studies show that lower utilization of maternity care services is observed among mothers who are over 35 years of age [11].

The results also indicated that an increase in the level of education of the women increases their likelihood of delivering in health facilities. These results are similar to other studies. Studies from Nepal have shown that the mother’s education being lower than primary level and not having had antenatal care is also associated with a high prevalence of home delivery without help of skilled birth attendants [10]. A higher education level, better awareness of health issues and an understanding of English were also significantly associated with facility delivery. Higher awareness of health issues was the factor that was significantly associated with facility delivery [12]. Also, more of those with no formal education and primary school education were the ones who chose to deliver at home compare to the others [13]. The issue of women’s education has been discussed at length in the context of health care seeking behaviour, and it would be reasonable to assume that it would have a positive effect on their own health. Studies show that maternal health education is consistently and strongly associated with all types of health behaviour and we expect use of maternal health care services to be higher among more educated mothers [11]. The decision making is thought to improve with higher level of education.

The results indicated that an increase in household income increases the likelihood of delivering in health facilities by 2.4 times. These results confirm other findings. Women of low socioeconomic status are less likely to use health facilities while those of higher status use the facilities. This status also affects affordability for services [10]. High income earners chose to deliver in the hospital more than low income earners (P<0.0010 [13]. Economic and physical accessibility are key factors that contribute to choice of facility. Households with a greater ability to pay are more likely to access delivery services outside the home [14]. Economic status is an important indicator of access to health care services [11]. The economic level determines the purchasing capacity of women for services including transport and hospital services.

The results indicated that an increase in pregnancies and parity reduces the likelihood of delivering in health facility. The findings are similar to other studies. Available literature shows that women who have more than three children are less likely to use skilled birth attendance during delivery [10]. Facility use is also higher among first and low-order births [14].

Parity, the number of children ever born, is strongly associated with health seeking behaviour. Studies show that primiparous women are consistently more likely to deliver with the assistance of a health professional than any other parity group. High parity women are the least likely to seek maternity care services due to greater confidence and cumulative experience. On the other hand, nulliparous women seek early maternity care services [11]. Attendance of four or more ante natal visits increases the likelihood of health facility delivery and early attendance of ante natal care increases the likelihood of delivering in health facility. If a woman made at least four ante natal visits, she was more likely to deliver in health facility [12]. Birth preparedness increases the likelihood of health facility delivery. These results are similar to studies that indicated that birth preparedness is one of the critical factors in determining the likelihood of having institutional delivery and checkups after delivery. Women, with individual birth plans have a greater likelihood of going for institutional deliveries that woman with no preparation [15]. In a study in the same area, the proportion with individual birth plans was low (20%) but among those with individual birth plans, the proportion of health facility deliveries was high [16]. The meaning of this study is that health facility deliveries is determined by specific factors that can be addressed if the uptake will be enhanced. This study had strength of being done in a rural area that displayed relevance because of constrains that exist in such environments. It had a weakness of being done in health facilities contrary to community set ups. This means some women may have been missed if they never came to the health facilities either for post natal care or child health clinics. Future studies should focus on community set ups and vulnerable populations like slums, refugees and teenagers.

## Conclusion

Health facility delivery is critical in the outcome of every pregnancy. Health facility deliveries have been associated with better outcomes than home deliveries. Majority (79%) of the respondents delivered in health facilities. The determinants of place of delivery are maternal age, level of education, household income, parity, attendance of ante natal care four or more times and birth preparedness. The study recommends that the ministry of health should upscale the antecare by sensitizing women on focused ante natal visits and birth preparedness. During antenatal visits strategies to be developed to ensure women with high parity and increased maternal age deliver in health facilities and under skilled birth attendance. A multisectoral approach should be undertaken to improve household incomes. The ministry of education should improve the level of education of girls which later increases the decision making capacity during years of child bearing.

### What is known about this topic

- The significance of health facility delivery in maternal health.

### What this study adds

- The study has helped to identify predictors of health facility delivery in rural areas. Health facility can be predicted by a variety of factors. An increase in the level of education, uptake of antenatal care, birth preparedness and household income increases the likelihood of a health facility delivery. To the contrary, an increase in parity decreases the likelihood of health facility delivery.
